# Audiovisual integrative training for augmenting cognitive- motor functions in older adults with mild cognitive impairment

**DOI:** 10.1186/s12877-020-1465-8

**Published:** 2020-02-17

**Authors:** Leung-Pong Lee, Afifah Wing-Yiu Har, Chun-Hei Ngai, Daniel W. L. Lai, Bess Yin-Hung Lam, Chetwyn Che-Hin Chan

**Affiliations:** 1 Department of Rehabilitation Sciences, The Hong Kong Polytechnic University, Kowloon, Hong Kong; 2 Department of Applied Social Sciences, The Hong Kong Polytechnic University, Kowloon, Hong Kong; 3 Institute of Active Ageing, The Hong Kong Polytechnic University, Kowloon, Hong Kong; 4 University Research Facility in Behavioral and Systems Neuroscience, The Hong Kong Polytechnic University, Kowloon, Hong Kong

**Keywords:** Attentional control, Audiovisual integration, Mild cognitive impairment, Upper limb motor functions

## Abstract

**Background:**

Previous studies indicated that the behavioral performances of older adults could be enhanced by multisensory integration. This pilot study tested the benefits of an audiovisual integrative (AV) training for improving the cognitive and upper limb motor functions in older adults with mild cognitive impairment (MCI).

**Methods:**

Twenty participants, according to their MoCA scores, with ten in each of a MCI (mean age = 63.3 years) and healthy older adult group (mean age = 64.7 years), engaged in AV integrative training. They were recruited from the Institute of Active Ageing at the Hong Kong Polytechnic University. The screening was conducted from February to March 2018 and the training program which consisted of three sessions (2 h each) was conducted from 14-28th May 2018. Their executive function, attention and upper limb functions were measured by the Stroop Test and Purdue Pegboard Test respectively.

**Results:**

The mixed linear model analysis results showed significant Time x Group interaction effects in the time used in the Stroop Test (dots) (*p* = 0.042) and the Purdue Pegboard scores (non-dominant hand use) (*p* = 0.025). The MCI group exhibited significantly more improvements in attentional control and non-dominant hand motor functions after the training.

**Conclusions:**

The findings suggest that the AV integrative training has the potential for enhancing the cognitive and motor functions of older adults with MCI. Furthermore, AV integrative training can serve as an alternative non-pharmacological intervention for combating neurodegeneration in older adults.

**Trial registration:**

This study has been retrospectively registered at the Chinese Clinical Trial Registry which is a World Health Organisation approved registry. Trial registration: Current Controlled Trials ChiCTR2000029408, January 29th, 2020.

## Background

Multisensory integration occurs when information from more than one sensory modality is perceived and synthesized [1]. Previous studies indicated that behavioral performances can be enhanced by multisensory integration [2]. In terms of the age effect on multisensory integration, it was found that older adults undergo significantly more enhancement in multi-sensory processing when compared to younger adults due to the effects of general cognitive slowing [3, 4]. On the other hand, in Mahoney et al. multisensory orienting cues significantly improved attention in both old and young adults [5]. Also, previous event-related potential (ERP) studies [6] revealed a super-additive effect when the congruent stimuli were encoded which is called audiovisual (AV) integration. For instance, Zuo and colleagues found that enhanced task performance was correlated with the audiovisual super-additive effect only in an older but not younger group [7]. They further observed that the super-additive effect was associated with the declined attention function of their older participants, which corresponds to the notion that older adults tend to voluntarily compensate for their age-related neurodegeneration as asserted by Grady [8]. Along the same line, another ERP study by Giard et al. suggested that multi-sensory integration which is mediated by a highly adaptive physiological processes occurrs very early in the sensory processing chain and operates in both sensory-specific and nonspecific cortical structures in different ways [9]. Moreover, Laurienti et al. reported that AV integration ameliorated age-related decline in cognitive functions such as attention [6]. Although these findings suggest that AV integration benefits older adults in different age-related outcomes, they have yet to be translated to intervention training programs for older adults.

In this study, we were interested in adapting the audiovisual integrative approach to design a training program for older adults, especially for those with mild cognitive impairment (MCI). According to the Mayo Clinic, MCI is the stage between expected normal neurodegeneration due to natural ageing and the more serious type of decline due to the pathology of dementia [10]. The decline in cognitive function is a common and early symptom in individuals with MCI, especially in the domains of attention, working memory and executive functions [11, 12]. Effective intervention has been commented to target at specific rather than general cognitive functions [13, 14]. The present study aimed at improving the attentional control of individuals with MCI using AV integrative training. In terms of the effect of multisensory integration on individuals with MCI, Wu et al. [15], found that patients with MCI and alzhiemer’s disease exhibited sufficient audiovisual integration, a greater peak (time bin with the highest percentage of benefit) and broader temporal window (time duration of benefit) of multisensory enhancement when compared to healthy older adults. This finding suggests that multi- sensory stimuli may offer a preferential performance benefit due to the adverse impact of aging on individual sensory systems. Based on previous finding, it is believed that older adults with MCI, who are associated with more sensory processing declines than healthy controls, might benefit more in their behavioral performances from multisensory integration which reduces their sensory processing demands. Therefore, the present study hypothesized enhanced attention and motor functions in the individuals with MCI than the healthy older adults after AV integrative training. Attentional control was selected as the main outcome measure mainly because it is affected more often and earlier than other cognitive functions. More importantly, its decline was reported to be related to the atrophy of the dorsolateral prefrontal cortex (DLPFC) which disrupts the integrative function of the frontal and parietal lobes [16–18]. This disruption has been characterized in the individuals with MCI [19, 20]. In addition to attentional control, upper limb motor function was also investigated in the present study because previous findings suggest that multisensory integration significantly enhances motor control and motor learning [21].

Although there are a number of non-pharmacological interventions to improve the cognitive decline in individuals with MCI including aerobic exercise, Chinese calligraphy, and mental activity [22–24], the current study was the first to adopt an audiovisual integrative approach for the individuals with MCI. Furthermore, it was to fill the knowledge gap of whether audiovisual integrative training would has a super-additive effect and be beneficial for enhancing cognitive and motor functions of older adults, especially to those with MCI. To address the literature gap, the present study aimed to adapt the AV integrative approach to examine the effectiveness of a training program to improve functions in older adults, specifically those with mild cognitive impairment (MCI) who have cognitive decline (e.g., attention and executive functions) [11]. It was hypothesized that older adults with MCI have more significant improvements in their attentional control and upper limb motor functions concurrently undergoing AV integrative training when compared with those healthy controls.

## Methods

### Subjects

One hundred and eighteen community dwelling older adults were screened between February and March in 2018. Among them, 72 participants aged above 60 years met the following inclusion criteria: (1) scored 18 to 25 on the Hong Kong version of the Montreal Cognitive Assessment (HK-MoCA) for the MCI group, and scored 26 or above for the healthy control group; and (2) intact finger dexterity measured by the Nine Hole Peg Test (within 22.9 s) [25] and visual acuity ability tested with the standard logarithmic visual acuity chart test (> 0.8). All participants did not receive musical instrument training for three months or longer and were not diagnosed with any major physical illness, mental disorder, or other disability. For this pilot study, only 20 participants were randomly selected from those 72 participants with 10 in MCI (mean age = 63.3 years) and healthy control group (mean age = 64.7 years) according to their MoCA scores [26]. The demographic characteristics and scores on different measures are displayed in Table 1.

**Table 1:** Demographic characteristics of the participants in the AV training for both MCI group and healthy control group

	Healthy control (*n* = 10)	MCI (*n* = 10)	*p*-value
Gender (%)			
Female	6 (60%)	7 (70%)	
Male	4 (40%)	3 (30%)	
Mean Age (SD)*	64.7 (3.2)	63.3 (2.7)	0.305
Mean Attendance (%) (SD)	100 (0)	100 (0)	
Mean Score on MoCA (SD)			
Mean Score (SD)*	27.3 (1.3)	24.2 (1)	< 0.001***
Minimum	26	22	
Maximum	30	25	
Mean Score on 9HPT (SD)*	19.7 (1.2)	18.6 (2)	0.133
Education (years) (SD)*	15.3 (4.8)	13.35 (4.1)	0.342

Note: MoCA, Montreal cognitive assessment; 9HPT, The nine-hole peg test; MoCA, Montreal cognitive assessment; *between-group comparisons were conducted using *t*-tests; ***p < =0.001, **p < 0.01, *p < 0.05

### Procedure

Selected participants were asked to complete three sessions of AV integrative training within a week from 14th to 28th May 2018. Each session lasted for 2 h (a total of 6 h for the entire AV integrative training). The Taiko video game was adopted for the AV integrative training in this study. Specifically, the participants learnt to play aTaiko drum (Japanese percussion instrument) according to the songs chosen through the Playstation 4 rhythm game entitled “Taiko no Tatsujin”. Noises were added to the visual and auditory stimuli given that the use of 100% clear stimuli might contribute to inverse effectiveness [27]. The input device used was a simulated electronic Taiko. This Taiko video game training involved hitting the drum with two drumsticks, one for each hand. Specifically, with each piece of chosen song playing in the background, the circular cues moving through a target zone were displayed on a computer screen. The participants were required to make precisely timed motor responses as accurately as possible by hitting the drum when the circular cues displayed on the screen fell into the target zone. If the cue displayed was red, then the participants had to hit the centre of the drum while the rim of the drum should be hit if the cue was blue. Training was administered and monitored by the occupational therapy trainees. Ethics approval was obtained from the Hong Kong Polytechnic University and written informed consent was obtained from each participant. The demographic information of the participants was collected and they were asked to complete the following assessments at the baseline and post-training.

### Measures

The Stroop Test was used to assess executive function, which also involves selective attention, processing speed and cognitive flexibility [28]. For the Stroop Test, there are three conditions: dots, words, color and words. The time that the participants took to finish each condition was recorded.

The Purdue Pegboard Test was used to assess the gross movement of the upper limbs, finger dexterity and bimanual coordination [29]. The participants were instructed to perform four separate tests with their dominant hand, non-dominant hand, both hands, and the assembly task.

### Statistical analysis

Independent t-tests were used to test the between-group differences for the demographic characteristics and performance on the outcome measures. Mixed linear models were used to analyse the effects of intervention. There were two types of mixed linear models performed in the present study. For each dependent measure in the assessments of pre−/post training, a 2 (Time: baseline and post intervention) × 2 (Group: MCI and healthy control) mixed linear model for repeated measures was conducted. For each dependent measure in task-related performance, a 3 (Time: after the 1st session, after the 2nd session, and after the 3rd session) × 2 (Group: MCI and healthy control) mixed linear model for repeated measures was also conducted. Multiple comparisons using paired *t*-tests were performed on those with significant interaction effects. The IBM SPSS v.25 was used for conducting the analyses, and the statistical significance was set as *p* ≤ 0.05*.*

## Results

Mixed linear models were performed with the outcome measures using IBM SPSS v.25 and the statistical significance was set as *p* ≤ 0.05*.* In terms of the treatment effects, significant Time x Group interaction effects were found in the time used in the Stroop Test (dots) (*p* = 0.042) and the Purdue Pegboard scores (non-dominant hand use) (*p* = 0.025) (Table 1). Significant Time x Group interaction effects were found for the Stroop Test (dots) (*F* (1, 18)=4.782, *p* = 0.042), in which the MCI group exhibited significant improvement from the baseline to the post-training (*p* = 0.006) while it was not significant for the healthy control group (Table 1). In terms of the Time x Group interaction effect, it was found in the task with non-dominant hand (*F* (1, 18) = 6.009, *p* = 0.025), in which the MCI group showed significant improvement after training (*p* = 0.004) (Table 2). No other significant interaction effects were found in other tasks in the Purdue Pegboard Test.

**Table 2:** Results of mixed linear model analyses for AV training outcome measures across Groups and Time

Outcomes	Mixed between-within ANOVA		
	Group^a^ *p*-value	Time^b^ *p*-value	Group x Time *p*-value
Stroop Test (Time (s))			
Dots	0.431	0.056	0.042*
Words	0.126	0.559	0.488
Colour and words	0.327	0.03*	0.719
Purdue pegboard			
Dominant	0.303	< 0.001***	0.85
Non-Dominant	0.478	0.003**	0.025*
Both	0.823	0.156	0.51
Assembly	0.498	< 0.001***	0.148

***p = < 0.001, **p < 0.01, *p < 0.05

^a^Groups: MCI group and healthy control group

^b^Time: Baseline and post-training

## Discussion

The main findings in the present study supported the hypotheses that the MCI participants would gain significantly more improvements than the healthy group in the attentional control ability as measured by the Stroop Test (dots) as well as the non-dominant hand upper limb function as measured by the Purdue Pegboard Test. The present findings are consistent with previous finding that more behavioral improvements were found in individuals with MCI and alzheimer’s disease when compared to healthy older adults via multisensory integration [15]. Specifically, the AV integrative training exposed the participants to encode both visual (blue or red circles) and audial (verbalization of red or blue) stimuli which operationalized audiovisual integration. The encoding of the stimuli and hence generation of the appropriate responses (hitting the drum) demanded strong attentional control and hand motor functions. Improvements in attentional control in the MCI group was supported by the significant increases in the scores on the dot subscale of the Stroop Test from the baseline to the post-training assessment. The Stroop Test (dots) is a measure of attentional control function [30], which was previously suggested to involve the left dorsolateral prefrontal cortex (DLPFC, Brodmann’s Area 9). The task demands of the AV integrative training are consistent with the characteristics of challenging attentional control [30]. In summary, the present findings support the notion that audiovisual integration is likely to benefit the individuals with MCI by way of improving their attention. Our findings are consistent with those revealed by Zou et al. that the older adults who showed greater gain in the superadditive process were found to have higher attention ability [7].

The significant improvements in attentional control but not in other cognitive domains in the MCI group are consistent with those revealed in other studies on percussion training for post-stroke patients. The reason might be because it is the precursor of other cognitive functions [13]. Besides attentional control, the AV integrative training was found to significantly enhance the MCI participants’ non-dominant hand function when compared to the healthy controls, which is consistent with prior findings [31]. It is noteworthy that the MCI group had a much lower score for the non-dominant hand than the healthy group, which was not the case for the dominant hand. The weaker non-dominant hand function among the MCI participants was attributed to the potential under-use of the hand. Our results are consistent with a previous study which employed transcranial direct current stimulation as the intervention and revealed that the non-dominant hemisphere appeared to be more responsive to the intervention [32]. This previous finding suggests that the cortical plasticity of the non-dominant hemisphere offers the potential for electrical stimulation to augment the function of the non-dominant hand.

### Limitations

The present study suffered from a number of limitations. First, since the same measures were used at the baseline and post-training which might induce a practice effect, the treatment effects might be contaminated. Future studies should use alternative measures at different time points of assessment to eliminate the practice effect. Second, the number of errors for the Stroop Test and Purdue Pegboard Test were not documented and these estimates should be recorded in future studies.

## Conclusions

In summary, the current findings support that the AV integrative training has the potential for enhancing the cognitive and motor functions of older adults with MCI. Importantly, the present findings suggest that AV integrative training can serve as an alternative non-pharmacological intervention for combating cognitive and motor decline in older adults.

## Data Availability

The datasets used and/or analysed during the current study are available from the corresponding author on reasonable request.

## Abbreviations

AV: Audiovisual
ERP: Event-related potential
HK-MoCA: The Hong Kong version of Montreal Cognitive Assessment
MCI: Mild cognitive impairment
